# Lipidemic Profile Changes over a Two-Year Intervention Period: Who Benefited Most from the Feel4Diabetes Program?

**DOI:** 10.3390/nu12123736

**Published:** 2020-12-04

**Authors:** Kalliopi Karatzi, George Moschonis, Eirini Botsi, Stavros Liatis, Kaloyan Tsochev, Pilar De Miguel-Etayo, Jemina Kivelä, Katja Wikström, Roumyana Dimova, Emese Antal, Itziar Lamiquiz-Moneo, Imre Rurik, Greet Cardon, Violeta Iotova, Konstantinos Makrilakis, Yannis Manios, on behalf of the Feel4Diabetes-Study Group

**Affiliations:** 1 Department of Nutrition and Dietetics, School of Health Science and Education, Harokopio University, 17671 Athens, Greece; pkaratzi@hua.gr (K.K.); eirhnh.botsh@gmail.com (E.B.); 2 Department of Dietetics, Nutrition and Sport, School of Allied Health, Human Services and Sport, La Trobe University, Melbourne, VIC 3086, Australia; g.moschonis@latrobe.edu.au; 3 National and Kapodistrian University of Athens Medical School, 11527 Athens, Greece; s.liatis@yahoo.com (S.L.); kmakrila@med.uoa.gr (K.M.); 4 Department of Paediatrics, Medical University Varna, 9002 Varna, Bulgaria; kalooyan@abv.bg (K.T.); violeta.iotova@mu-varna.bg (V.I.); 5 Growth, Exercise, Nutrition and Development (GENUD) Research Group, Instituto Agroalimentario de Aragón (IA2), Instituto de Investigación Sanitaria de Aragón (IIS Aragón), Universidad de Zaragoza, 50009 Zaragoza, Spain; pilardm@unizar.es; 6 Centro de Investigación Biomédica en Red Enfermedades Cardiovasculares (CIBERCV), Instituto de Salud Carlos III, 28029 Madrid, Spain; 7 Department of Public Health Solutions, Finnish Institute for Health and Welfare, 00271 Helsinki, Finland; jemina.kivela@thl.fi (J.K.); katja.wikstrom@thl.fi (K.W.); 8 Department of Diabetology, Clinical Center of Endocrinology, Medical University Sofia, 1431 Sofia, Bulgaria; dr.roumyana.dimova@gmail.com; 9 Hungarian Society of Nutrition, 1088 Budapest, Hungary; diet.Emese.Antal@gmail.com (E.A.); rurik.imre@med.unideb.hu (I.R.); 10 Hospital Universitario Miguel Servet, Instituto de Investigación Sanitaria Aragón (IIS Aragón), CIBERCV, Universidad de Zaragoza, 50009 Zaragoza, Spain; itziarlamiquiz@gmail.com; 11 Department of Movement and Sports Sciences, Faculty of Medicine and Health Sciences, Ghent University, 9000 Gent, Belgium; Greet.Cardon@UGent.be

**Keywords:** Feel4Diabetes study, lifestyle intervention, lipidemic profile

## Abstract

Identification of participants’ characteristics who benefited most from large community-based intervention studies may guide future prevention initiatives in order to maximize their effectiveness. The current study aimed to examine the socio-demographic, anthropometric, and behavioral characteristics, as well as the health and eating perceptions of those who improved their lipidemic profile, in the Feel4Diabetes early screening and prevention program. In the present analyses, 1773 adults from families at high risk for developing type 2 diabetes mellitus (T2DM) were enrolled, receiving either the standard care or the more intensive intervention, and 33.3–55.2% of them improved one or more of their lipidemic indices by >5%. Women, people living in Southeastern Europe, coming from two-parent families, having higher financial security, educational level and better diet quality were associated with a 27–64% higher likelihood for benefiting from the program regarding one or more of their lipidemic profile indices. Participants who were overweight or obese (especially with central obesity), employed, with prolonged sedentary behavior, prone to emotional eating and perceiving their weight status as lower than their actual weight were 24–43% less likely to have benefited. These findings should guide future interventions, prioritizing regions in greater need, and being tailor-made to specific population characteristics in order to further improve their effectiveness.

## 1. Introduction

Dyslipidemia and insulin resistance, as well as unhealthy lifestyle behaviors (i.e., poor diet quality, increased sedentary time, abnormal body weight, smoking) have long been identified as risk factors for cardiovascular disease (CVD) development [1]. Dyslipidemia is a major CVD risk factor for both healthy subjects and patients with type 2 diabetes mellitus (T2DM), accounting for 72–85% of diabetics, but it is also very common in insulin resistant subjects who do not yet have established T2DM [2,3]. Therefore, a large amount of people at risk of T2DM also have dyslipidemia, experiencing a double burden regarding CVD manifestation.

According to the World Health Organization, all mentioned risk factors are modifiable, and over 75% of all CVD mortality may be prevented by effective interventions delivering adequate lifestyle changes [1,4]. In Europe, CVD prevention has been a priority since the late 1990s and its focus has been on promotion of a heart healthy lifestyle and management of risk factors, especially in people at high risk for CVD like people with prediabetes or T2DM [5]. Consequently, large-scale randomized controlled trials (RCTs) were conducted using various lifestyle interventions in populations at high risk of developing T2DM, aiming to reduce the rate of T2DM development, as well as to ameliorate risk factors such as dyslipidemia that are associated with both T2DM and CVD morbidity and mortality [6].

There is great variability regarding behavioral intervention frequency and intensity, as well as inconsistency regarding the populations that would benefit most from these interventions, which makes the optimum strategy for the delivery of lifestyle programs unclear [6,7,8,9,10,11]. These findings raise concern regarding people that receive less benefit from these interventions, and characteristics of these people have not yet been efficiently studied. Yet, the need for screening the population in order to identify and highlight the main characteristics of those who benefit most from behavioral interventions is a crucial step regarding the improvement of future prevention strategies in order to maximize their efficacy, to be cost effective, and to be successfully implemented in broader population groups.

Due to the need to prioritize the most vulnerable groups and to perform early screening for identifying high risk families, the Feel4Diabetes (F4D) program was designed and implemented in six EU member states, comprised of a community-based screening phase and an intervention phase providing either standard care or a more intensive intervention component. Therefore, the aim of the present study was to examine the sociodemographic, anthropometric, lifestyle and behavioral characteristics of those who benefited most, regarding their lipidemic profile, from the Feel4Diabetes program.

## 2. Materials and Methods

### 2.1. Study Design and Sampling Procedures

The Feel4Diabetes study [12] (National Clinical Trial number, NCT 02393872) was a large school and community-based intervention implemented over two years (2016–2018) that aimed to promote a healthy lifestyle, including healthy eating and enhancing physical activities, in order to alleviate the negative outcomes of obesity and obesity-related metabolic risk factors in vulnerable families in Europe.

The study was conducted within selected provinces in six European countries, representing low income countries (Bulgaria and Hungary), high income countries (Belgium and Finland) and countries under austerity measures (Greece and Spain). Recruitment was based on a standardized, multi-stage sampling procedure targeting vulnerable population groups at high risk for developing type 2 diabetes mellitus (T2DM). In Bulgaria and Hungary (i.e., low/middle-income countries (LMICs)), all the municipalities within the selected regions were eligible for recruitment, while in Belgium, Finland, Greece and Spain (i.e., high income countries (HICs)), families within low socioeconomic status (SES) municipalities were recruited. Low SES municipalities were defined as those with the lowest educational level and/or the highest unemployment rates, based on official resources and local authorities, within each country. More details regarding study design can be found elsewhere [13,14].

In brief, primary schools located in the selected municipalities were used as the entry point to the community, targeting families with children in the first three grades of primary education. Afterwards, they were invited to go through a self-reported, screening procedure with the use of the Finnish Diabetes Risk Score (FINDRISK) questionnaire, answering brief questions like their age, fruit consumption and family history of diabetes in order to identify the “high risk families” based on the risk possibility of developing T2DM [15]. Families with at least one “high risk parent” (having a high FINDRISK score) were defined as “high risk families” and the adult members of these families were invited to go through a brief medical check-up at local community centers. Depending the municipality they were living in (intervention or control municipality), they either received standard care or a more intensive intervention. Standard care comprised of a brief medical check-up and one counselling session for the adult members of the families annually. The intensive intervention was comprised of six counselling sessions in the first year (beyond the annual medical check-ups) and motivational feedback and guidance via Short Message Service (SMS) intervention in the second year. Moreover, a school-based intervention was delivered for all families in the intervention municipalities. More details regarding the study design and the screening procedures followed can be found elsewhere [13,14]. In the present study, 1330 families were included, out of which 24.7% and 25.4% were adult members of the same family in the more intensive intervention and standard care arm, respectively.

### 2.2. Ethical Approvals and Consent Forms

The Feel4Diabetes study adhered to the Declaration of Helsinki and the conventions of the Council of Europe on human rights and biomedicine. Prior to initiating the intervention, all participating countries obtained ethical clearance from the relevant ethical committees and local authorities. More details regarding the approvals received from each country can be found elsewhere [13,14].

### 2.3. Measurements

All measurements were conducted by trained research assistants using standardized procedures and calibrated portable equipment.

#### 2.3.1. Anthropometry

Height was measured without shoes and was recorded to the nearest tenth of a centimeter (i.e., 0.1 cm) using telescopic stadiometers: SECA 213, SECA 214, SECA 217 and SECA 225 (SECA International). Body weight was measured with light clothing and without shoes and recorded to the nearest 0.1 kg. The equipment included electronic weight scales: SECA 813 and SECA 877. Body mass index (BMI) cut-off points were used for the classification of all volunteers according to their body weight. Waist Circumference (WC) measurements were recorded to the nearest tenth of centimeter (i.e., 0.1 cm) using a non-elastic measuring tape (SECA 201) (SECA International) and the World Health Organization (WHO) cut-off points were used for their classification [16].

#### 2.3.2. Blood Test

Blood samples were taken by qualified staff in the morning after overnight fasting (for at least 8 h). Total cholesterol (TC), High-density lipoprotein cholesterol (HDL-C) and triglycerides (TG) levels were measured with an enzymatic colorimetric test (Roche Diagnostics SA, Vasilia, Switzerland) in an automatic analyzer (Roche/Hitachi Modular). Low-density lipoprotein cholesterol (LDL-C) was calculated using the Friedewald formula [17]. The National Cholesterol Education Program (NCEP) was used in order to detect any dyslipidemia cases [18]. TC/HDL-C, LDL-C/HDL-C and the atherogenic index of plasma (AIP) were also calculated. AIP was calculated using the formula: AIP = log 10 (TG/HDL-C) [19].

#### 2.3.3. Healthy Diet Score

Information regarding dietary habits was self-reported via standardized questionnaires. Participants reported dietary habits including main meals (breakfast, lunch, dinner) and snacking, as well as the frequency of consumption of particular food items (i.e., dairy, bread, fruits, vegetables, pulses, meat, fish, salty snacks, nuts/seeds, tea and coffee, soft drinks with or without sugar and alcoholic beverages) using a semi-quantitative food frequency questionnaire.

Collected data regarding dietary habits were used to calculate the Healthy Diet Score (HDS), which was developed and validated within the F4D study and consisted of components with high importance as risk or protective factors for T2DM. This score is based on 12 main dietary components including breakfast, vegetables, fruit and berries, sugary drinks, whole-grain cereals, nuts and seeds, low-fat dairy products, oils and fats, red meat, sweet snacks, salty snacks and family meals. The total score ranges from 0–100, with higher scores indicating better dietary quality [17].

#### 2.3.4. Socio-Demographic and Behavioral Characteristics

Information regarding basic demographic and socio-economic characteristics (e.g., paternal and maternal years of education and age), as well as health and eating perceptions of the families participating in the Feel4Diabetes program, was collected using standardized questionnaires in all study participants. Behavioral indices regarding drinking, eating, physical activity and sedentary behaviors, as well as their determinants, were self-reported via standardized questionnaires. All questionnaires were developed, validated and standardized between the participating countries before the study.

#### 2.3.5. Statistical Analysis

Continuous variables are presented as means ± standard deviations and categorical values as proportions (%). The normality of distribution of variables was determined by the Kolmogorov–Smirnov test and histograms.

Participants were identified as benefiting from participating either to the more intensive intervention or to the standard care treatment if either their total cholesterol, LDL, HDL, triglycerides levels, total cholesterol/HDL, LDL/HDL or AIP in the first or second year was >5% better than at baseline. Cut-off points of 5% were based on literature showing that even a minimum reduction in LDL cholesterol levels, if sufficiently extended over time, enable cardiovascular risk reduction [20,21].

Logistic regression analyses were employed to examine associations between benefiting from the intervention (dependent variable) and participant characteristics (independent variables). Analyses were adjusted for baseline age and baseline values of the dependent variable (i.e., total cholesterol, LDL) (continuous), sex, region (categorical) and intervention arm (categorical). Statistical analysis was performed using the Statistical Package for Social Sciences (SPSS Inc., Chicago, IL, USA), version 25.0. The level of statistical significance was set at *p* <  0.05.

## 3. Results

In the present study, 1773 adults from families with a high risk of developing T2DM and with full data at baseline were included in the analysis. Their mean age at baseline was 40.8 ± 5.7 years; their BMI was 28.6 ± 5.5 kg/m^2^ and their LDL cholesterol was 120.9 ± 33.2 mg/dL. As depicted in Table 1, 33.3–55.2% of the participants improved >5% for one or more indices of their lipidemic profile after the Feel4Diabetes first year intervention period.

The odds for benefiting from either the more intensive intervention or the standard care treatment regarding a >5% improvement in either total, LDL and HDL cholesterol, triglycerides, total cholesterol/HDL, LDL/HDL and AIP are shown in Table 2 and Table 3. Women, people living in Southeastern Europe, those in two-parent families, those with higher income and educational level, and having better dietary quality have a 27–64% higher probability of benefiting from the intervention (i.e., altering their blood lipid indices by >5%). On the other hand, employed people, those with prolonged sedentary behavior, being overweight or obese (especially those with central obesity), those who choose food emotionally and those who perceive their weight status as lower than their actual weight had a 22–43% lower probability of benefiting from the intervention.

## 4. Discussion

The present study aimed to identify the sociodemographic, anthropometric, lifestyle and behavioral characteristics of the participants experiencing the most benefit regarding their lipidemic profile as a consequence of the Feel4Diabetes program (i.e., “more intensive intervention” or “standard care”). It was shown that women, those living in Southeastern Europe, two-parent families, with higher financial security and educational level and better diet quality have a higher probability of benefiting from the intervention, while employed people, those with prolonged sedentary behavior, being overweight or obese (especially those with central obesity), those prone to emotional eating and those perceiving their body weight as lower than their actual weight had a lower probability of benefitting from the intervention. These findings further highlight the knowledge regarding population groups that experience difficulties in benefiting from an intervention targeting behavioral and lifestyle changes. These findings could facilitate the design of cost-effective future intervention programs that are tailor-made to the needs of specific population subgroups in order to enable T2DM and CVD prevention among those in higher need.

The present findings reveal that the Feel4Diabetes program was successful in facilitating several lifestyle changes in order to reduce the risk of developing T2DM, and to control other risk factors like dyslipidemia. This is evidenced by the current analyses which showed that 33.3–55.2% of the study participants benefited by improving one or more biochemical indices regarding their lipidemic profile. This could be attributed to the screening procedure and the intervention delivered, which was guided by existing knowledge from previous intervention studies, with the aim to adjust as much as possible to the individual characteristics of people from different countries and cultures [13]. Interestingly, the intervention did not follow the “one size fits all” approach, but it enabled countries, schools and families to prioritize their goals from a pre-selected list of targeted energy balance-related behaviors (EBRBs) identified from the literature. Also, countries were able to adjust their behavioral change messages, delivered by the counselling sessions, according to their unique cultural characteristics. However, the standardized protocols and procedures followed for implementing the program across all centers and the central training of all research team members safeguard an accurate and reliable assessment and increase the validity of the study findings.

However, not all study participants benefited from the Feel4Diabetes program. People with a lower probability of benefitting from the intervention were men, people living in Northern Europe, people of a lower educational level and lower income, those with poor dietary choices and prolonged sedentary time and those who were overweight or obese, yet they underestimate their weight status and emotionally choose their food. Low-income and low-educated people have been described to have a higher prevalence of T2DM and major CVD risk factors like dyslipidemia and obesity than people of higher socioeconomic status (SES) [22,23,24]. It has already been described that social factors, including low income and low educational level, have a direct association with poor dietary choices such as choosing low cost-foods (which are usually energy-dense and nutrient poor), as well as with unhealthy lifestyle behaviors (like low physical activity, increased sedentary time and smoking), leading to adverse consequences to their metabolic health [25]. Also, accumulating evidence supports that the obesity epidemic has led to misconceptions regarding healthy body weight status and body image [26]. An interesting explanation regarding the underlying cause for weight status underestimation is the theory of “visual normalization”, where the common presence of larger body sizes has caused a recalibration to the range of body sizes perceived as normal [26]. Underestimation of excess body weight might inhibit the adoption of healthy weight-related attitudes and behaviors among overweight or obese individuals and could further increase the risk of T2DM and CVD development [27]. Regarding the influence of region of residence, it was shown that people living in Southeastern Europe present with a higher probability of benefitting by improving their lipidemic profile. This finding may be related to the existing differences between Central/North and Southeast Europe, with the later having less facilities in terms of social support and community resources, road and personal insecurity, limited access to community facilities (e.g., sports halls, parks, pedestrian areas), as well as a less developed primary care and prevention system. This usually leads to poor adherence to dietary and physical activity recommendations [28], yet the present findings show that people in Southeastern Europe have a greater need for such prevention programs, with higher intervention effectiveness than in Central/Northern Europe. Also, possible genetic differences cannot be disregarded.

These findings confirm the need to increase the effectiveness of large prevention programs in certain population groups. Existing knowledge has shown that there is a wide gap between knowledge and management around behavioral interventions. This has led to implementation challenges or uncertainty as to the most effective approach targeting specific population subgroups, which often require costly and intense interventions. However, several such programs have experienced high dropout rates [29]. Yet, successful interventions like Feel4Diabetes, which are well designed and tailor-made in order to appeal to the majority of the population studied, should be further adjusted appropriately in order to sufficiently motivate the most vulnerable and hard-to-reach groups of participants in any future prevention initiations.

In line with these thoughts for future prevention programs is that in most analyses performed, both arms of the Feel4Diabetes program (either the standard care or the more intensive intervention component) presented with almost the same probability of benefiting the study participants in terms of their lipid profile. Interestingly, in regard to the first year’s results of HDL level changes, it was shown that standard care was even better than the more intensive intervention component. As future interventions should be cost-effective, it should be noticed that even one counselling session was enough to benefit the participants and that one counselling session was as effective as seven. Therefore, it is possible that not only the intervention itself, but also the screening of people is highly capable to motivate them in order to alter their lifestyle behaviors. Therefore, future public health initiatives aiming to tackle T2DM should carefully consider the vulnerable population groups identified in the present study, starting from the early identification of people, and be easy-to-apply, relatively low-cost and potentially scalable and sustainable in order to be transferred to the wider population [14]. What is most important is that perhaps face-to-face counselling should be kept at a minimum for offering standard care to the participants, and possibly the use of new technologies and smart phone applications should be implemented in order to instantly motivate individuals to improve their dietary choices and increase their physical activity [30,31].

The study should be interpreted under the light of its strengths and limitations. One significant strength is that it is a very large intervention study conducted with more than 12,000 primary-school-aged children and their families, including more than 2000 families at high risk for developing type 2 diabetes, from six European countries. Also, the standardized protocols and procedures followed across all centers, the objectively collected data regarding biochemical and anthropometric indices, and the clinical and lifestyle data safeguard the assessment as more objective and reliable and increase the generalizability of the findings. The presented data are very important, as for the first time, several characteristics, perceptions and beliefs were revealed as possible barriers to the effectiveness of a large early screening and prevention program such as the Feel4Diabetes program, which will be used as the first step for future preventive programs to focus on people most in need for such initiatives who usually fail to take advantage of the benefits of such programs. On the other hand, part of the collected data was self-reported. Although the validity and reliability of the relevant questionnaires were tested before the start of the intervention, this approach is prone to recall bias and social desirability.

## 5. Conclusions

The Feel4Diabetes study, as a large early screening and prevention program, was found to be more effective regarding lipidemic profile changes at the end of the two-year intervention among women, people living in Southeastern Europe, coming from two-parent families, and those with higher financial security, higher educational level and better diet quality. Yet, participants who were overweight or obese (especially with central obesity), employed, with prolonged sedentary behavior, prone to emotional eating and perceiving their weight status as lower than their actual weight were less likely to benefit from this intervention. It is evident that due to economic differences among European regions, as well as due to the less developed primary care in Southeastern Europe, the need for and potentially the effectiveness of such initiatives is of primary importance in those regions. The present findings can guide the design and implementation of future prevention initiatives in order to promote easy-to-apply, efficient and cost-effective programs reaching even the most vulnerable and deprived population groups.

## Figures and Tables

**Table 1:** Characteristics of study participants at baseline.

	Total (*n* = 1773)
**Age (years)**	40.8 (5.7)
**Sex**	
**Males (%)**	35.0
**Females (%)**	65.0
**Region**	
**Northern Europe (%)**	29.3
**Southeastern Europe (%)**	70.7
**Education**	
**<12 years of education (%)**	25.3
**>12 years of education (%)**	74.7
**Anthropometrics**	
**BMI (kg/m^2^)**	28.6 (5.5)
**Waist circumference**	
**Males (cm)**	103.6 (12.0)
**Females (cm)**	90.2 (13.5)
**Health behaviors**	
**Screen time (h/day)**	3.6 (1.8)
**MVPA (min/day)**	15.2 (39.3)
**Healthy Diet Score**	49.2 (12.6)
**Current smokers (%)**	26.1
**Biochemical indices**	
**Total cholesterol (mg/dL)**	195.1 (37.9)
**LDL cholesterol (mg/dL)**	120.9 (33.2)
**HDL cholesterol (mg/dL)**	53.1 (14.2)
**Triglycerides (mg/dL)**	112.9 (96.5)
**Total cholesterol/HDL**	3.9 (1.3)
**LDL/HDL**	2.5 (0.9)
**AIP**	0.3 (0.3)
**Proportion of participants benefiting at the first year of intervention for:**	
**Total cholesterol (%)**	33.3
**LDL cholesterol (%)**	39.6
**HDL cholesterol (%)**	37.6
**Triglycerides (%)**	40.1
**Total cholesterol/HDL (%)**	37.4
**LDL/HDL (%)**	41.5
**AIP (%)**	53.2

Variables are presented as mean (SD) or %. BMI: body mass index, MVPA: moderate to vigorous physical activity, LDL: low density lipoprotein, HDL: high density lipoprotein, AIP: atherogenic index of plasma.

**Table 2:** Multivariable adjusted odds ratios (95% CI) of benefiting from the Feel4Diabetes program in the first and second year as defined by >5% improvement in total cholesterol, LDL cholesterol, HDL cholesterol and triglycerides.

	TOTAL CHOLESTEROL		LDL CHOLESTEROL		HDL CHOLESTEROL		TRIGLYCERIDES	
	Odds of Benefiting in First Year OR, 95% CI	Odds of Benefiting in Second Year OR, 95% CI	Odds of Benefiting in First Year OR, 95% CI	Odds of Benefiting in Second Year OR, 95% CI	Odds of Benefiting in First Year OR, 95% CI	Odds of Benefiting in Second Year OR, 95% CI	Odds of Benefiting in First Year OR, 95% CI	Odds of Benefiting in Second Year OR, 95% CI
**Group**								
**More intensive intervention**	1	1	1	1	1	1	1	1
**Standard care**	0.92 (0.74, 1.13)	1.00 (0.79, 1.26)	0.90 (0.73, 1.11)	1.09 (0.86, 1.37)	**1.28 (1.05, 1.58)**	1.03 (0.81, 1.32)	1.01 (0.82, 1.23)	1.18 (0.94, 1.48)
**Age (years)**								
**<45**	1	1	1	1	1	1	1	1
**>45**	0.84 (0.66, 1.07)	0.90 (0.69, 1.17)	0.88 (0.69, 1.11)	1.06 (0.82, 1.38)	1.15 (0.91, 1.46)	1.16 (0.88, 1.53)	0.82 (0.64, 1.04)	0.79 (0.61, 1.02)
**Sex**								
**Male (%)**	1	1	1	1	1	1	1	1
**Female (%)**	1.04 (0.82, 1.32)	1.16 (0.90, 1.50)	1.02 (0.81, 1.29)	1.24 (0.95, 1.61)	**1.36 (1.06, 1.74)**	**1.35 (1.01, 1.81)**	**1.36 (1.06, 1.74)**	1.16 (0.98, 1.52)
**Region**								
**Central/Northern** **Europe (%)**	1	1	1	1	1	1	1	1
**Southeastern Europe (%)**	1.22 (0.97, 1.53)	1.21 (0.94, 1.57)	**1.28 (1.02, 1.61)**	1.10 (0.85, 1.42)	1.25 (0.10, 1.56)	1.02 (0.78, 1.34)	0.96 (0.78, 1.20)	**1.42 (1.11, 1.83)**
**Education**								
**<12 years of education (%)**	1	1	1	1	1	1	1	1
**>12 years of education (%)**	0.80 (0.61, 1.05)	1.32 (0.97, 1.80)	0.90 (0.69, 1.18)	1.05 (0.77, 1.43)	1.13 (0.86, 1.48)	0.83 (0.60, 1.16)	1.11 (0.85, 1.46)	**1.43 (1.04, 1.95)**
**Occupation**								
**Unemployed**	1	1	1	1	1	1	1	1
**Employed**	**0.72 (0.56, 0.94)**	0.89 (0.66, 1.19)	**0.76 (0.59, 0.98)**	0.91 (0.68, 1.23)	1.07 (0.82, 1.40)	0.92 (0.66, 1.27)	0.84 (0.65, 1.09)	0.91 (0.68, 1.23)
**Marital status**								
**One-parent families**	1	1	1	1	1	1	1	1
**Two-parent families**	1.01 (0.66, 1.56)	0.95 (0.59, 1.54)	1.01 (0.67, 1.53)	0.96 (0.59, 1.56)	1.01 (0.66, 1.54)	1.03 (0.62, 1.70)	1.06 (0.70, 1.60)	1.70 (1.03, 2.79)
**Income status**								
**It is difficult to cover my expenses**	1	1	1	1	1	1	1	1
**It is easy to cover my expenses**	1.06 (0.84, 1.33)	1.02 (0.79, 1.31)	0.89 (0.71, 1.11)	0.91 (0.71, 1.17)	1.06 (0.85, 1.33)	1.22 (0.93, 1.59)	1.03 (0.83, 1.28)	**1.42 (1.11, 1.83)**
**Anthropometrics**								
**BMI**								
**<25 kg/m^2^**	1	1	1	1	1	1	1	1
**>25 kg/m^2^**	0.94 (0.74, 1.20)	0.96 (0.74, 1.26)	0.88 (0.69, 1.11)	0.88 (0.67, 1.14)	**0.75 (0.59, 0.96)**	0.83 (0.61, 1.12)	0.87 (0.69, 1.11)	0.77 (0.59, 1.01)
**Waist circumference**								
**<80 cm F, <94 cm M**	1	1	1	1	1	1	1	1
**>80 cm F, >94 cm M**	0.78 (0.56, 1.00)	1.02 (0.77, 1.36)	**0.76 (0.59, 0.97)**	0.90 (0.68, 1.20)	**0.57 (0.46, 0.78)**	**0.70 (0.51, 0.96)**	0.78 (0.60, 1.01)	0.79 (0.54, 1.05)
**Health behaviors**								
**Screen time**								
**<2 h/day**	1	1	1	1	1	1	1	1
**>2 h/day**	0.85 (0.68, 1.06)	1.01 (0.79, 1.29)	0.93 (0.75, 1.15)	0.91 (0.71, 1.16)	1.24 (1.01, 1.54)	0.86 (0.66, 1.11)	1.04 (0.84, 1.28)	0.85 (0.67, 1.08)
**MVPA**								
**<60 min/day**	1	1	1	1	1	1	1	1
**>60 min/day**	0.94 (0.71, 1.25)	**0.67 (0.48, 0.91)**	0.94 (0.72, 1.24)	0.79 (0.58, 1.09)	1.17 (0.89, 1.53)	1.15 (0.83, 1.60)	0.94 (0.72, 1.22)	0.92 (0.67, 1.25)
**Healthy Diet Score**								
**<Fourth quartile**	1	1	1	1	1	1	1	1
**>Fourth quartile**	1.07 (0.81, 1.42)	0.93 (0.68, 1.27)	1.12 (0.85, 1.46)	0.93 (0.68, 1.28)	**1.50 (1.15, 1.96)**	1.33 (0.96, 1.84)	1.03 (0.79, 1.35)	0.79 (0.58, 1.07)
**Smoking**								
**Non- or former smokers**	1	1	1	1	1	1	1	1
**Current smokers**	1.00 (0.77, 1.30)	0.94 (0.71, 1.26)	1.04 (0.81, 1.34)	0.88 (0.65, 1.18)	1.03 (0.80, 1.33)	0.78 (0.57, 1.07)	0.98 (0.76, 1.26)	0.79 (0.59, 1.05)
**Health and eating perceptions**								
**I believe health is determined by destiny**								
**Disagree**	1	1	1	1	1	1	1	1
**Agree**	1.21 (0.81, 1.80)	0.86 (0.55, 1.36)	1.09 (0.74, 1.61)	1.11 (0.71, 1.74)	0.97 (0.65, 1.44)	1.21 (0.76, 1.93)	0.96 (0.65, 1.43)	0.83 (0.52, 1.31)
**I have little power for disease prevention**								
**Disagree**	1	1	1	1	1	1	1	1
**Agree**	0.94 (0.67, 1.32)	0.90 (0.62, 1.31)	0.83 (0.59, 1.15)	1.01 (0.70, 1.47)	0.82 (0.59, 1.15)	0.80 (0.53, 1.22)	1.11 (0.80, 1.55)	0.73 (0.50, 1.08)
**I choose food without thinking**								
**Disagree**	1	1	1	1	1	1	1	1
**Agree**	0.94 (0.73, 1.22)	0.95 (0.72, 1.26)	0.83 (0.65, 1.08)	0.87 (0.65, 1.16)	0.92 (0.71, 1.18)	0.85 (0.63, 1.15)	0.93 (0.72, 1.19)	1.00 (0.76, 1.32)
**Weight status perception**								
**Accurate weight status perception**	1	1	1	1	1	1	1	1
**Weight status is perceived as lower than actual weight**	0.85 (0.66, 1.10)	1.03 (0.77, 1.38)	**0.75 (0.59, 0.97)**	0.96 (0.72, 1.28)	**0.72 (0.56, 0.93)**	0.91 (0.66, 1.25)	0.97 (0.76, 1.25)	1.02 (0.76, 1.36)

Benefit represents >5% reduction in either total, LDL cholesterol or triglycerides blood levels or >5% increase in HDL cholesterol levels in the first or second year of intervention. Analyses were adjusted (except when used as an independent variable) for baseline age (continuous), sex, region (categorical), intervention arm (categorical) and baseline values of the dependent variable. The bold indicator highlights the statistically significant findings. OR: Odds Ratio; C.I: Confidence Interval; HDL: High Density Lipoprotein; LDL: Low Density Lipoprotein; BMI: body mass index, MVPA: moderate to vigorous physical activity

**Table 3:** Multivariable adjusted odds ratios (95% CI) of benefiting from the Feel4Diabetes program in the first and second year as defined by a >5% improvement in total cholesterol/HDL, LDL cholesterol/HDL or AIP (log (triglycerides/HDL)).

	TOTAL CHOLESTEROL/HDL		LDL CHOLESTEROL/HDL		AIP	
	Odds of Benefiting in First Year OR, 95% CI	Odds of Benefiting in Second Year OR, 95% CI	Odds of Benefiting in First Year OR, 95% CI	Odds of Benefiting in Second Year OR, 95% CI	Odds of Benefiting in First Year OR, 95% CI	Odds of Benefiting in Second Year OR, 95% CI
**Group**						
**More intensive intervention**	1	1	1	1	1	1
**Standard care**	1.10 (0.90, 1.36)	0.98 (0.77, 1.25)	1.07 (0.87, 1.31)	1.06 (0.84, 1.35)	1.11 (0.92, 1.34)	1.15 (0.93, 1.43)
**Age (years)**						
**<45**	1	1	1	1	1	1
**>45**	1.10 (0.86, 1.39)	1.16 (0.88, 1.52)	1.02 (0.80, 1.29)	1.02 (0.78, 1.34)	1.01 (0.81, 1.27)	1.17 (0.91, 1.49)
**Sex**						
**Males (%)**	1	1	1	1	1	1
**Females (%)**	1.04 (0.80, 1.34)	1.11 (0.83, 1.49)	0.98 (0.77, 1.26)	1.03 (0.78, 1.36)	**1.27 (1.00, 1.61)**	**1.31 (1.01, 1.71)**
**Region**						
**Central/Northern** **Europe (%)**	1	1	1	1	1	1
**Southeastern Europe (%)**	**1.64 (1.30, 2.07)**	1.25 (0.95, 1.64)	**1.57 (1.25, 1.96)**	1.00 (0.77, 1.30)	1.05 (0.85, 1.29)	0.97 (0.77, 1.22)
**Education**						
**<12 years of education (%)**	1	1	1	1	1	1
**>12 years of education (%)**	1.02 (0.77, 1.33)	1.10 (0.79, 1.52)	0.87 (0.67, 1.14)	1.07 (0.78, 1.47)	1.27 (0.99, 1.63)	**1.33 (1.00, 1.78)**
**Occupation**						
**Unemployed**	1	1	1	1	1	1
**Employed**	0.89 (0.68, 1.16)	0.84 (0.61, 1.15)	0.95 (0.73, 1.23)	0.86 (0.63, 1.17)	0.86 (0.67, 1.10)	1.02 (0.78, 1.35)
**Marital status**						
**One-parent families**	1	1	1	1	1	1
**Two-parents families**	1.14 (0.74, 1.75)	0.85 (0.52, 1.40)	1.25 (0.82, 1.91)	0.81 (0.50, 1.31)	1.35 (0.91, 2.00)	**1.62 (1.03, 2.54)**
**Income status**						
**It is difficult to cover my expenses**	1	1	1	1	1	1
**It is easy to cover my expenses**	1.02 (0.81, 1.28)	1.09 (0.84, 1.42)	0.93 (0.74, 1.16)	1.04 (0.81, 1.35)	1.16 (0.94, 1.43)	**1.37 (1.08, 1.74)**
**Anthropometrics**						
**BMI**						
**<25 kg/m^2^**	1	1	1	1	1	1
**>25 kg/m^2^**	0.88 (0.69, 1.13)	0.87 (0.65, 1.17)	0.83 (0.65, 1.06)	0.85 (0.64, 1.13)	**0.62 (0.49, 0.78)**	**0.72 (0.56, 0.93)**
**Waist circumference**						
**<80 cm F, <94 cm M**	1	1	1	1	1	1
**>80 cm F, >94 cm M**	**0.72 (0.55, 0.95)**	0.78 (0.57, 1.07)	**0.76 (0.59, 0.98)**	0.75 (0.56, 1.01)	**0.63 (0.49, 0.80)**	0.80 (0.61, 1.06)
**Health behaviors**						
**Screen time**						
**<2 h/day**	1	1	1	1	1	1
**>2 h/day**	1.00 (0.80, 1.24)	0.86 (0.67, 1.11)	1.08 (0.87, 1.34)	0.87 (0.68, 1.12)	**0.76 (0.62, 0.93)**	0.88 (0.70, 1.10)
**MVPA**						
**<60 min/day**	1	1	1	1	1	1
**>60 min/day**	0.96 (0.72, 1.26)	**0.67 (0.48, 0.96)**	0.92 (0.70, 1.20)	0.78 (0.56, 1.09)	0.53 (0.92, 1.19)	0.89 (0.67, 1.18)
**Healthy Diet Score**						
**<Fourth quartile**	1	1	1	1	1	1
**>Fourth quartile**	1.20 (0.91, 1.58)	1.33 (0.96, 1.84)	1.12 (0.86, 1.47)	1.30 (0.95, 1.79)	0.93 (0.72, 1.19)	0.86 (0.65, 1.15)
**Smoking**						
**Non- or former smokers**	1	1	1	1	1	1
**Current smokers**	1.06 (0.82, 1.37)	0.95 (0.70, 1.30)	0.93 (0.72, 1.20)	0.82 (0.61, 1.12)	1.00 (0.79, 1.27)	0.78 (0.59, 1.02)
**Health and eating perceptions**						
**I believe health is determined by destiny**						
**Disagree**	1	1	1	1	1	1
**Agree**	0.99 (0.66, 1.48)	1.13 (0.71, 1.79)	1.04 (0.70, 1.54)	1.46 (0.934, 2.28)	1.08 (0.74, 1.56)	1.00 (0.66, 1.53)
**I have little power for disease prevention**						
**Disagree**	1	1	1	1	1	1
**Agree**	0.71 (0.50, 1.01)	0.84 (0.56, 1.26)	0.98 (0.71, 1.37)	0.97 (0.66, 1.42)	0.97 (0.71, 1.32)	0.73 (0.51, 1.04)
**I choose food without thinking**						
**Disagree**	1	1	1	1	1	1
**Agree**	**0.76 (0.59, 0.99)**	0.93 (0.69, 1.26)	0.82 (0.64, 1.06)	1.05 (0.79, 1.40)	0.86 (0.68, 1.09)	0.95 (0.73, 1.23)
**Weight status perception**						
**Accurate weight status perception**	**1**	1	1	1	1	1
**Weight status is perceived as lower than actual weight**	**0.75 (0.58, 0.97)**	0.93 (0.68, 1.27)	**0.74 (0.58, 0.95)**	0.91 (0.67, 1.23)	**0.78 (0.61, 0.99)**	0.78 (0.59, 1.01)

Benefit represents a >5% reduction in either total/HDL cholesterol, LDL/HDL blood levels or AIP at the first or second year of intervention. Analyses were adjusted (except when used as an independent variable) for baseline age (continuous), sex, region (categorical), intervention arm (categorical) and baseline values of the dependent variable. The bold indicator highlights the statistically significant findings. OR: Odds Ratio; C.I: Confidence Interval; HDL: High Density Lipoprotein; LDL: Low Density Lipoprotein; AIP: Atherogenic index of plasma; BMI: body mass index, MVPA: moderate to vigorous physical activity
